# Exploiting the Immunogenic Potential of Cancer Cells for Improved Dendritic Cell Vaccines

**DOI:** 10.3389/fimmu.2015.00663

**Published:** 2016-01-14

**Authors:** Lien Vandenberk, Jochen Belmans, Matthias Van Woensel, Matteo Riva, Stefaan W. Van Gool

**Affiliations:** ^1^Laboratory of Pediatric Immunology, Department of Immunology and Microbiology, KU Leuven University of Leuven, Leuven, Belgium; ^2^Laboratory of Experimental and Neuroanatomy, Department of Neurosciences, KU Leuven University of Leuven, Leuven, Belgium; ^3^Laboratory of Pharmaceutics and Biopharmaceutics, Université Libre de Bruxelles, Brussels, Belgium; ^4^Department of Neurosurgery, San Gerardo Hospital, University of Milano-Bicocca, Monza, Italy; ^5^Kinderklinik, RWTH, Aachen, Germany; ^6^Immunologic-Oncologic Centre Cologne (IOZK), Köln, Germany

**Keywords:** immunotherapy, dendritic cell vaccines, immunogenic cell death, antitumor immunity, tumor lysate, immunogenicity

## Abstract

Cancer immunotherapy is currently the hottest topic in the oncology field, owing predominantly to the discovery of immune checkpoint blockers. These promising antibodies and their attractive combinatorial features have initiated the revival of other effective immunotherapies, such as dendritic cell (DC) vaccinations. Although DC-based immunotherapy can induce objective clinical and immunological responses in several tumor types, the immunogenic potential of this monotherapy is still considered suboptimal. Hence, focus should be directed on potentiating its immunogenicity by making step-by-step protocol innovations to obtain next-generation Th1-driving DC vaccines. We review some of the latest developments in the DC vaccination field, with a special emphasis on strategies that are applied to obtain a highly immunogenic tumor cell cargo to load and to activate the DCs. To this end, we discuss the effects of three immunogenic treatment modalities (ultraviolet light, oxidizing treatments, and heat shock) and five potent inducers of immunogenic cell death [radiotherapy, shikonin, high-hydrostatic pressure, oncolytic viruses, and (hypericin-based) photodynamic therapy] on DC biology and their application in DC-based immunotherapy in preclinical as well as clinical settings.

## Introduction

Cancer immunotherapy has gained considerable momentum over the past 5 years, owing predominantly to the discovery of immune checkpoint inhibitors. These inhibitors are designed to release the brakes of the immune system that under physiological conditions prevent auto-immunity by negatively regulating cytotoxic T lymphocyte (CTL) function. Following the FDA approval of the anti-cytotoxic T lymphocyte-associated antigen-4 (CTLA-4) monoclonal antibody (mAb) ipilimumab (Yervoy) in 2011 for the treatment of metastatic melanoma patients (1), two mAbs targeting programed death (PD)-1 receptor signaling (nivolumab and pembrolizumab) have very recently joined the list of FDA-approved checkpoint blockers (respectively, for the treatment of metastatic squamous non-small cell lung cancer and relapsed/refractory melanoma patients) (2, 3).

However, the primary goal of cancer immunotherapy is to activate the immune system in cancer patients. This requires the induction of tumor-specific T-cell-mediated antitumor immunity. Checkpoint blockers are only able to abrogate the brakes of a functioning antitumoral immune response, implying that only patients who have pre-existing tumor-specific T cells will benefit most from checkpoint blockade. This is evidenced by the observation that ipilimumab may be more effective in patients who have pre-existing, albeit ineffective, antitumor immune responses (4). Hence, combining immune checkpoint blockade with immunotherapeutic strategies that prime tumor-specific T cell responses might be an attractive and even synergistic approach. This relatively new paradigm has lead to the revival of existing, and to date disappointing (as monotherapies), active immunotherapeutic treatment modalities. One promising strategy to induce priming of tumor-specific T cells is dendritic cell (DC)-based immunotherapy.

Dendritic cells are positioned at the crucial interface between the innate and adaptive immune system as powerful antigen-presenting cells capable of inducing antigen-specific T cell responses (5). Therefore, they are the most frequently used cellular adjuvant in clinical trials. Since the publication of the first DC vaccination trial in melanoma patients in 1995, the promise of DC immunotherapy is underlined by numerous clinical trials, frequently showing survival benefit in comparison to non-DC control groups (6–8). Despite the fact that most DC vaccination trials differ in several vaccine parameters (i.e., site and frequency of injection, nature of the DCs, choice of antigen), DC vaccination as a monotherapy is considered safe and rarely associates with immune-related toxicity. This is in sharp contrast with the use of mAbs or cytokine therapies. Ipilumumab has, for instance, been shown to induce immune-related serious adverse events in up to one-third of treated melanoma patients (1). The FDA approval of Sipuleucel-T (Provenge), an autologous DC-enriched vaccine for hormone-resistant metastatic prostate cancer, in 2010 is really considered as a milestone in the vaccination community (9). After 15 years of extensive clinical research, Sipileucel-T became the first cellular immunotherapy ever that received FDA approval, providing compelling evidence for the substantial socio-economic impact of DC-based immunotherapy. DC vaccinations have most often been applied in patients with melanoma, prostate cancer, high-grade glioma, and renal cell cancer. Although promising objective responses and tumor-specific T cell responses have been observed in all these cancer-types (providing proof-of-principle for DC-based immunotherapy), the clinical success of this treatment is still considered suboptimal (6). This poor clinical efficacy can in part be attributed to the severe tumor-induced immune suppression and the selection of patients with advanced disease status and poor survival prognostics (6, 10–12).

There is a consensus in the field that step-by-step optimization and standardization of the production process of DC vaccines, to obtain a Th1-driven immune response, might enhance their clinical efficacy (13). In this review, we address some recent DC vaccine adaptations that impact DC biology. Combining these novel insights might bring us closer to an ideal DC vaccine product that can trigger potent CTL- and Th1-driven antitumor immunity.

One factor requiring more attention in this production process is the immunogenicity of the dying or dead cancer cells used to load the DCs. It has been shown in multiple preclinical cancer models that the methodology used to prepare the tumor cell cargo can influence the *in vivo* immunogenic potential of loaded DC vaccines (14–19). Different treatment modalities have been described to enhance the immunogenicity of cancer cells in the context of DC vaccines. These treatments can potentiate antitumor immunity by inducing immune responses against tumor neo-antigens and/or by selectively increasing the exposure/release of particular damage-associated molecular patterns (DAMPs) that can trigger the innate immune system (14, 17–19). The emergence of the concept of immunogenic cell death (ICD) might even further improve the immunogenic potential of DC vaccines. Cancer cells undergoing ICD have been shown to exhibit excellent immunostimulatory capacity owing to the spatiotemporally defined emission of a series of critical DAMPs acting as potent danger signals (20, 21). Thus far, three DAMPs have been attributed a crucial role in the immunogenic potential of nearly all ICD inducers: the surface-exposed “eat me” signal calreticulin (ecto-CRT), the “find me” signal ATP and passively released high-mobility group box 1 (HMGB1) (21). Moreover, ICD-experiencing cancer cells have been shown in various mouse models to act as very potent Th1-driving anticancer vaccines, already in the absence of any adjuvants (21, 22). The ability to reject tumors in syngeneic mice after vaccination with cancer cells (of the same type) undergoing ICD is a crucial hallmark of ICD, in addition to the molecular DAMP signature (21).

Here, we review the effects of three frequently used immunogenic modalities and four potent ICD inducers on DC biology and their application in DC vaccines in preclinical as well as clinical settings (Tables 1 and 2). Moreover, we discuss the rationale for combining different cell death-inducing regimens to enhance the immunogenic potential of DC vaccines and to ensure the clinical relevance of the vaccine product.

**Table 1:** **A list of prominent enhancers of immunogenicity and ICD inducers applied in DC vaccine setups and their associations with DAMPs and DC biology**.

Treatment modality	Associated DAMPs	Effect on DC biology
**Immunogenic treatment modality**		
UV irradiation	Pre-apoptotic ecto-CRT (23); post-apoptotic passive release of HSP70 and HMGB1 (24); mutation-induced neo-antigens (25)	Efficient engulfment; phenotypic maturation; increased IL-12 secretion; stimulate the polarization of T cells toward CTLs (19, 24, 26, 27)

Oxidation-inducing modalities (HOCl/H_2_O_2_ treatment or freeze–thaw cycles followed by X-ray irradiation)	OAMPs (reactive protein carbonyls, peroxidized phospholipids, oxidized low-density lipoprotein) (14, 18, 28–30); carbonylated protein products presented as neo-antigens (30, 31)	Efficient antigen uptake and presentation; induction of IL-12; increased *in vivo* induction of tumor-reactive T cells (14); induction of Th1- and CTL-driven antitumor immunity (18)

Heat shock	Passive release of heat shock proteins like HSP60/70/90 (17, 32); passive release of HMGB1 (33); increased expression of tumor-specific antigens (34)	Upregulation of DC maturation markers (CD40, CD80, and CD86) and induction of IL-12 (32); enhanced priming of CTL responses (17, 34)

**Inducers of immunogenic cell death**		
Radiotherapy	Pre-apoptotic exposure of ecto-CRT (23, 24, 35); early/mid-apoptotic exposure of ecto-HSP70 (36); post-apoptotic passive release of HMGB1 (33, 35); mutation-induced neo-antigens (25)	Efficient phagocytosis and enhanced phenotypic maturation (37); increased infiltration in the tumor environment (38, 39); enhanced stimulation of antigen-specific CTL responses (40)

Shikonin	Early/mid-apoptotic induction of ecto-HSP70, ecto-CRT and ecto-GRP78 (an inducer of pro-tumorigenic effects) (41)	Increased phenotypic (CD40^high^, CD80^high^, CD86^high^) and functional maturation (IL-12p70^high^, TGF-β^high^, IL-6^high^, IL-23^low^) but only in combination with LPS; increased capacity to induce Th1 and Th17 differentiation (41)

High-hydrostatic pressure	Early/mid-apoptotic exposure of ecto-HSP70, ecto-HSP90, ecto-CRT; pre-apoptotic ATP release; post-apoptotic passive release of HMGB1, HSP70/90, and CRT (42)	Efficient phagocytosis; enhanced phenotypic and functional maturation; induction of antigen-specific T cells without inducing Tregs (42)

Oncolytic viruses	CVB3 and oncolytic adenovirus: (early-apoptotic) exposure of ecto-CRT; (early/mid-apoptotic) secretion of ATP and (post-apoptotic) release of HMGB1 (43, 44)	Enhanced expression of CD80/CD86 (44, 46, 47) and CCR7 (44); more efficient priming of tumor-specific CD8^+^ CTL responses (43, 46, 47) and Th1 responses (43); increased accumulation in tumor microenvironment (43, 44)
	NDV: early/mid-necroptotic exposure of ecto-CRT and post-necroptotic release of HMGB1 (45)	

Hypericin-based PDT	Pre-apoptotic ecto-CRT, ecto-HSP70 and secreted ATP; late apoptotic passive release of HSP70/90, CRT and HMGB1; accumulation of OAMPs like protein carbonyls (48–50)	Enhanced phagocytosis; phenotypic maturation (CD80^high^ CD86^high^ CD83^high^ MHC-II^high^) and immunogenic functional stimulation (NO^high^ IL-10^absent^ IL-6^high^ IL-1β^high^ IL-12p70^medium^); clonal expansion of human IFN-γ producing CD4^+^ and CD8^+^ T cells (49, 53, 54)

Photofrin-based PDT	early/mid-apoptotic exposure of CRT, HSP60/70, ceramide and S1P; post-apoptotic release of HMGB1 (51, 52)	Increased phenotypic maturation (CD86^high^, MHC-II^high^) and enhanced IL-12 production (55); increased infiltration in tumor draining lymph nodes after peritumoral vaccination (56)

*CRT,calreticulin; CTL, cytotoxic T lymphocyte; CVB3, coxsackievirus B3; DAMPs, damage-associated molecular patterns; HMGB1, high-mobility group box-1 protein; HSP, heat shock protein; ICD, immunogenic cell death; IFN, interferon; LPS, lipopolysaccharide; NDV, Newcastle disease virus; NO, nitric oxide; OAMPs, oxidation-associated molecular patterns; PDT, photodynamic therapy; TGF, transforming growth factor; Treg, regulatory T cell*.

**Table 2:** **A list of preclinical tumor models and clinical studies for evaluation of the *in vivo* potency of DC vaccines loaded with immunogenically killed tumor cells**.

Treatment modality	Preclinical experience in DC vaccine settings	Clinical experience in DC vaccine settings
**Immunogenic treatment modalities**		
UV irradiation	B16 melanoma in C57BL/6 – curative immunizations (19); ID8-ova ovarian carcinoma model in C57BL/6 mice – weekly curative immunizations (14)	Only in combination with γ-irradiation and heat shock in B-cell lymphoma patients (57)

Oxidation-inducing modalities (HOCl/H_2_O_2_ treatment or freeze–thaw cycles followed by X-ray irradiation)	ID8-ova ovarian carcinoma model in C57BL/6 mice – weekly curative immunizations (14); orthotopic GL261 high-grade glioma model in C57BL/6 mice – both prophylactic and curative vaccination settings induced a pro-inflammatory shift in the brain-infiltrating immune cells and the protein carbonyl content in the tumor lysate positively correlated with tumor rejection (18)	Freeze–thaw cycles in combination with high-dose irradiation: often reported in clinical trials involving high-grade glioma and melanoma patients (8, 58–66)
		HOCl: pilot study in five recurrent ovarian cancer patients demonstrated potent T cell responses against tumor antigens, decreased circulating Treg levels, and serum IL-10 levels and two patients experienced durable PFS responses of ≥24 months (14)

Heat shock	PANCO2 pancreatic cancer model in C57BL/6 mice – curative vaccinations (17); in combination with 30 Gy irradiation in B16-ova model in C57BL/6 mice – prophylactic vaccinations (16)	Non-randomized trial in newly diagnosed glioblastoma patients (67): significantly improved tumor control rates and survival rates in DC vaccine group than in control group; increased proportions of peripheral CD4^+^ and CD8^+^ T cells post vaccination compared to control group; in combination with other cell killing modalities in B-cell lymphoma and melanoma patients (57, 68)

**Inducers of immunogenic cell death**		
Radiotherapy	B16 melanoma in C57BL/6 – prophylactic immunization model with critical involvement of CD4^+^ and CD8^+^ T cells (15, 37); E.G7 (SCCVII) in C57BL/6 – curative vaccination model (40)	Radiotherapy as a single intervention: multiple clinical trials in melanoma patients (8) and two clinical trials in high-grade glioma patients (69, 70). This study by Cho and colleagues reported a survival advantage of more than 15 months in the vaccinated glioblastoma patients in comparison to the control group (receiving conventional treatment)
		Radiotherapy as part of an ICD-inducing cell death protocol in B-cell lymphoma patients (57)

Shikonin	B16 melanoma in C57BL/6 – curative immunization model with strong induction of CTL responses (41)	Not available

High-hydrostatic pressure	Preclinical experiments are currently ongoing (71)	Multiple clinical trials are initiated involving prostate and ovarian cancer patients (71)

Oncolytic viruses	Not applied as ICD-based DC vaccines yet; curative combination of intratumoral oncolytic virus treatment and peripheral DC vaccination in B16 melanoma (C57BL/6) (72) and in subcutaneous CMT64 or KNL205 tumors (in C57BL/6 mice and DBA/2 DREG mice, respectively) (73)	Case report of breast cancer patient treated with combination of local hyperthermia, intravenously administered NDV and intradermal DC vaccines loaded with NDV-oncolysate (74)

Hypericin-based PDT	Not available	Not available
Photofrin-based PDT	*In vivo* photofrin-PDT treatment in combination with curative DC vaccination in C-26 colon carcinoma (BALB/c) (75); curative vaccinations with DCs charged with PDT-induced tumor lysate in EMT6, Renca and 4T1 non-orthotopic tumor modes (BALB/c), induction of CTL and Th1 responses	Not available

*CRT, calreticulin; CTL, cytotoxic T lymphocyte; DC, dendritic cell; ICD, immunogenic cell death; NDV, Newcastle disease virus; PDT, photodynamic therapy; PFS, progression-free survival*.

## The Impact of DC Biology on the Efficacy of DC Vaccines

Over the past years, different DC vaccine parameters have been shown to impact the clinical effectiveness of DC vaccinations. In the next section, we will elaborate on some promising adaptations of the DC preparation protocol.

Given the labor-intensive *ex vivo* culturing protocol of monocyte-derived DCs and inspired by the results of the Provenge study, several groups are currently exploiting the use of blood-isolated naturally circulating DCs (76–78). In this context, De Vries et al. evaluated the use of antigen-loaded purified plasmacytoid DCs for intranodal injection in melanoma patients (79). This strategy was feasible and induced only very mild side effects. In addition, the overall survival of vaccinated patients was greatly enhanced as compared to historical control patients. However, it still remains to be determined whether this strategy is more efficacious than monocyte-derived DC vaccine approaches (78). By contrast, experiments in the preclinical GL261 high-grade glioma model recently showed that vaccination with tumor antigen-loaded myeloid DCs resulted in more robust Th1 responses and a stronger survival benefit as compared to mice vaccinated with their plasmacytoid counterparts (80).

In view of their strong potential to stimulate cytotoxic T cell responses, several groups are currently exploring the use of Langerhans cell-like DCs as sources for DC vaccines (81–83). These so-called IL-15 DCs can be derived from CD14^+^ monocytes by culturing them with IL-15 (instead of the standard IL-4). Recently, it has been shown that in comparison to IL-4 DCs, these cells have an increased capacity to stimulate antitumor natural killer (NK) cell cytotoxicity in a contact- and IL-15-dependent manner (84). NK cells are increasingly being recognized as crucial contributors to antitumor immunity, especially in DC vaccination setups (85, 86). Three clinical trials are currently evaluating these Langerhans cell-type DCs in melanoma patients (NCT00700167, NCT 01456104, and NCT01189383).

Targeting cancer stem cells is another promising development, particularly in the setting of glioma (87). Glioma stem cells can foster tumor growth, radio- and chemotherapy-resistance, and local immunosuppression in the tumor microenvironment (87, 88). Furthermore, glioma stem cells may express higher levels of tumor-associated antigens and MHC complex molecules as compared to non-stem cells (89, 90). A preclinical study in a rodent orthotopic glioblastoma model has shown that DC vaccines loaded with neuropsheres enriched in cancer stem cells could induce more immunoreactivity and survival benefit as compared to DCs loaded with GL261 cells grown under standard conditions (91). Currently there are four clinical trials ongoing in high-grade glioma patients evaluating this approach (NCT00890032, NCT00846456, NCT01171469, and NCT01567202).

With regard to the DC maturation status of the vaccine product, a phase I/II clinical trial in metastatic melanoma patients has confirmed the superiority of mature antigen-loaded DCs to elicit immunological responses as compared to their immature counterparts (92). This finding was further substantiated in patients diagnosed with prostate cancer and recurrent high-grade glioma (93, 94). Hence, DCs need to express potent costimulatory molecules and lymph node homing receptors in order to generate a strong T cell response. In view of this finding, the route of administration is another vaccine parameter that can influence the homing of the injected DCs to the lymph nodes. In the context of prostate cancer and renal cell carcinoma it has been shown that vaccination routes with access to the draining lymph nodes (intradermal/intranodal/intralymphatic/subcutaneous) resulted in better clinical response rates as compared to intravenous injection (93). In melanoma patients, a direct comparison between intradermal vaccination and intranodal vaccination concluded that, although more DCs reached the lymph nodes after intranodal vaccination, the melanoma-specific T cells induced by intradermal vaccination were more functional (95). Furthermore, the frequency of vaccination can also influence the vaccine’s immunogenicity. Our group has shown in a cohort-comparison trial involving relapsed high-grade glioma patients that shortening the interval between the four inducer DC vaccines improved the progression-free survival curves (58, 96).

Another variable that has been systematically studied is the cytokine cocktail that is applied to mature the DCs. The current gold standard cocktail for DC maturation contains TNF-α, IL-1β, IL-6, and PGE_2_ (97, 98). Although this cocktail upregulates DC maturation markers and the lymph node homing receptor CCR7, IL-12 production by DCs could not be evoked (97, 98). Nevertheless, IL-12 is a critical Th1-driving cytokine and DC-derived IL-12 has been shown to associate with improved survival in DC vaccinated high-grade glioma and melanoma patients (99, 100). Recently, a novel cytokine cocktail, including TNF-α, IL-1β, poly-I:C, IFN-α, and IFN-γ, was introduced (101, 102). The type 1-polarized DCs obtained with this cocktail produced high levels of IL-12 and could induce strong tumor-antigen-specific CTL responses through enhanced induction of CXCL10 (99). In addition, CD40-ligand (CD40L) stimulation of DCs has been used to mature DCs in clinical trials (100, 103). Binding of CD40 on DCs to CD40L on CD4^+^ helper T cells licenses DCs and enables them to prime CD8^+^ effector T cells.

A final major determinant of the vaccine immunogenicity is the choice of antigen to load the DCs. Two main approaches can be applied: loading with selected tumor antigens (tumor-associated antigens or tumor-specific antigens) and loading with whole tumor cell preparations (13). The former strategy enables easier immune monitoring, has a lower risk of inducing auto-immunity, and can provide “off-the-shelf” availability of the antigenic cargo. Whole tumor cell-based DC vaccines, on the other hand, are not HLA-type dependent, have a reduced risk of inducing immune-escape variants, and can elicit immunity against multiple tumor antigens. Meta-analytical data provided by Neller et al. have demonstrated enhanced clinical efficacy in several tumor types of DCs loaded with whole tumor lysate as compared to DCs pulsed with defined tumor antigens (104). This finding was recently also substantiated in high-grade glioma patients, although this study was not set-up to compare survival parameters (105).

## Toward a More Immunogenic Tumor Cell Cargo

The majority of clinical trials that apply autologous whole tumor lysate to load DC vaccines report the straightforward use of multiple freeze–thaw cycles to induce primary necrosis of cancer cells (8, 93). Freeze–thaw induced necrosis is, however, considered non-immunogenic and has even been shown to inhibit toll-like receptor (TLR)-induced maturation and function of DCs (16). To this end, many research groups have focused on tackling this roadblock by applying immunogenic modalities to induce cell death.

### Immunogenic Treatment Modalities

**Figure 1:**
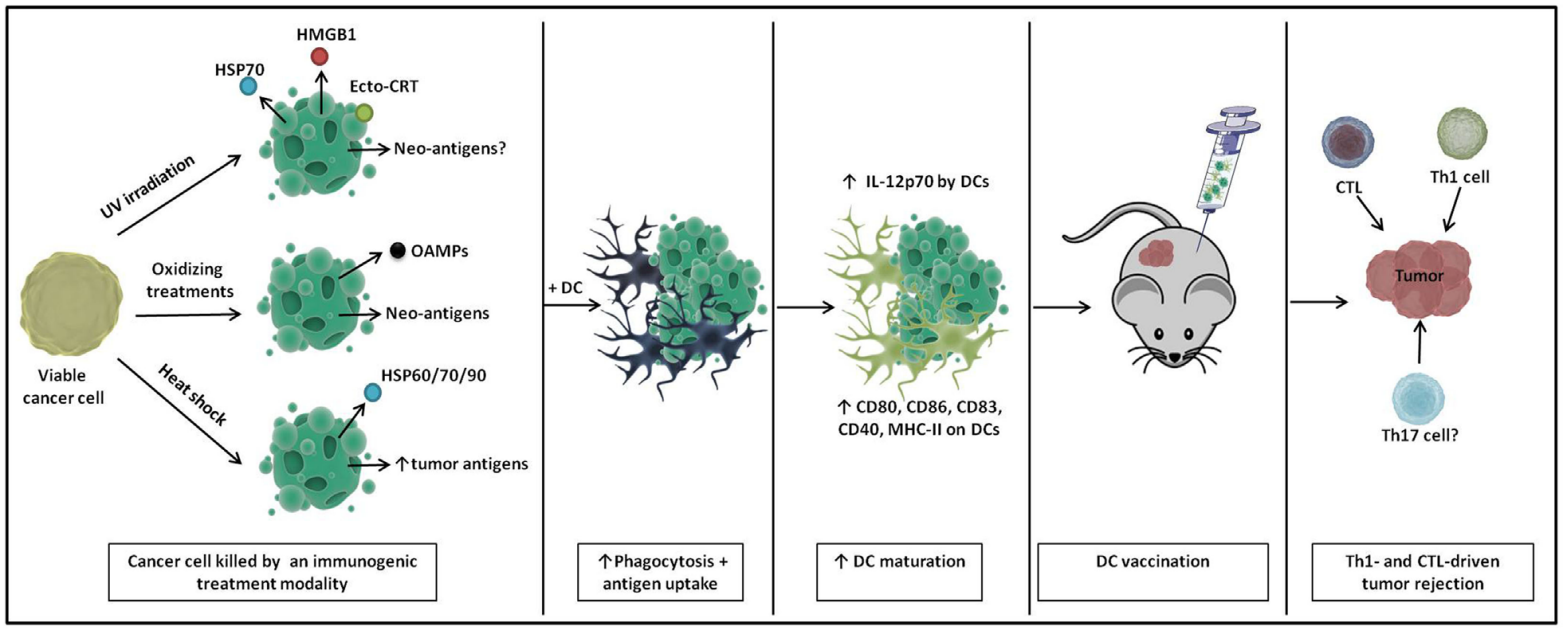
**A schematic representation of immunogenic DC vaccines**. Cancer cells show enhanced immunogenicity upon treatment with UV irradiation, oxidizing treaments, and heat shock, characterized by the release of particular danger signals and the (increased) production of tumor (neo-)antigens. Upon loading onto DCs, DCs undergo enhanced phagocytosis and antigen uptake and show phenotypic and partial functional maturation. Upon *in vivo* immunization, these DC vaccines elicit Th1- and cytotoxic T lymphocyte (CTL)-driven tumor rejection.

Tables 1 and 2 list some frequently applied treatment methods to enhance the immunogenic potential of the tumor cell cargo that is used to load DC vaccines in an ICD-independent manner (i.e., these treatments do not meet the molecular and/or cellular determinants of ICD). Immunogenic treatment modalities can positively impact DC biology by inducing particular DAMPs in the dying cancer cells (Table 1). Table 2 lists the preclinical and clinical studies that investigated their *in vivo* potential. Figure 1 schematically represents the application and the putative modes of action of these immunogenic enhancers in the setting of DC vaccines.

#### Ultraviolet Irradiation

Ultraviolet (UV) light is considered an electromagnetic non-ionizing radiation with a wavelength between 100 and 400 nm. Its immunogenic potential was discovered in 1991 when Begovic et al. demonstrated that vaccination of immunocompetent mice (but not immunodeficient nude mice) with UV-irradiated cancer cells could induce resistance to subsequent rechallenge with live tumor cells (23, 106, 107). This antitumor effect was crucially mediated by NK cells and CD8^+^ T cells. UV-treated cancer cells are efficiently engulfed by DCs, leading to phenotypic maturation and increased IL-12 production (19, 24, 26) (Table 1). Moreover, these matured DCs in turn stimulated the polarization of T cells toward IFN-γ producing CD8^+^ T cells (24, 26). Of note, human DCs that had ingested UV-irradiated apoptotic tumor cells were shown to be more effective in generating CD8^+^ CTLs than DCs pulsed with freeze–thaw lysates (27). In addition, immunization with DCs loaded with UV-treated tumor cells could elicit effective antitumor therapeutic efficacy in a B16 mouse melanoma model, albeit non-superior to DCs loaded with necrotic freeze–thaw lysate (19) (Table 2). The induction of specific DAMPs, such as ecto-CRT, and the release of heat shock protein 70 (HSP70) and HMGB1 determines the immunogenicity of UV irradiation (23, 24, 33) (Table 1). Moreover, as UV light is known to affect mainly DNA, mutation-induced tumor neo-antigens might also contribute to increasing the host antitumor immune response (108). T cells reactive against mutated neo-antigens are theoretically less susceptible to central and peripheral tolerance. Vaccination with UV-induced tumor neo-antigens might be particularly useful in UV-induced tumors (e.g., cutaneous and uveal melanoma) that might share the *ex vivo* UV-induced tumor neo-antigens. Besides, it has previously been shown that immunization of tumor-bearing mice with mutated melanoma-derived self-antigens can elicit efficient cross-reactive CD8^+^ T cell responses against multiple non-mutated epitopes of the tumor protein and against the melanoma cells (109). This led to the rejection of established poorly immunogenic B16 melanoma tumors (109). To the best of our knowledge, there are no reports of clinical trials that used UV irradiation as a single treatment for obtaining an antigen source to pulse DC vaccines (Table 2). This is probably related to the fact that UV light as a single treatment is not able to induce high levels of cancer cell death in the vaccine, an absolute requirement for clinical translation.

#### Oxidation-Inducing Modalities

In recent years, an increasing number of data were published concerning the ability of oxidative stress to induce oxidation-associate molecular patterns (OAMPs), such as reactive protein carbonyls and peroxidized phospholipids, which can act as DAMPs (28, 29) (Table 1). Protein carbonylation, a surrogate indicator of irreversible protein oxidation, has for instance been shown to improve cancer cell immunogenicity and to facilitate the formation of immunogenic neo-antigens (30, 31).

One prototypical enhancer of oxidation-based immunogenicity is radiotherapy (21, 23). In certain tumor types, such as high-grade glioma and melanoma, clinical trials that apply autologous whole tumor lysate to load DC vaccines report the random use of freeze–thaw cycles (to induce necrosis of cancer cells) or a combination of freeze–thaw cycles and subsequent high-dose γ-irradiation (8, 18) (Table 2). However, from the available clinical evidence, it is unclear which of both methodologies has superior immunogenic potential. In light of the oxidation-based immunogenicity that is associated with radiotherapy, we recently demonstrated the superiority of DC vaccines loaded with irradiated freeze–thaw lysate (in comparison to freeze–thaw lysate) in terms of survival advantage in a preclinical high-grade glioma model (18) (Table 2). This survival advantage was associated with an increased tumor infiltration of Th1 cells and CTLs and accompanied by a reduced invasion of regulatory cells (Tregs), macrophages, and myeloid-derived suppressor cells. Moreover, this study revealed a significant positive correlation between the level of protein carbonylation – as a measure of the total oxidative content – in the tumor lysates used to load the DCs and the percentage of mice able to reject the aggressive intracranial tumors. Treatment of the tumor lysate with hydrogen peroxide (H_2_O_2_, a strong oxidant) even induced higher tumor protection than irradiated freeze–thaw lysate, warranting the preclinical investigation of other strong oxidizing modalities to further potentiate the immunogenicity of whole tumor antigen-pulsed DC vaccinations.

In line with these results and through a series of elegant *ex vivo* an *in vivo* mouse experiments, Chiang et al. recently selected hypochlorous acid (HOCl)-based oxidation (to induce primary necrosis of tumor cells) as the method of choice (as compared to UVB irradiation and freeze–thaw cycles) for preparing whole tumor lysate-loaded DC vaccines in the preclinical ID8 ovarian cancer model (14) (Table 2). Interestingly, T cells stimulated by DCs loaded with HOCl-induced oxidatively modified tumor cells were still able to recognize non-modified tumor cells, an essential requirement if the cells are to exert antitumor activity (30). In a pilot study containing five recurrent ovarian cancer patients, these autologous DCs loaded with HOCl-oxidized autologous tumor lysate could produce high levels of IL-12, elicited strong antigen-specific T cell responses and reduced the levels of circulating Tregs and serum IL-10 (14). Moreover, two patients experienced durable progression-free survival intervals of more than 24 months after vaccination (Table 2).

#### Heat Shock Treatment

Heat shock is a term that is applied when a cell is subjected to a temperature that is higher than that of the ideal body temperature of the organisms of which the cell is derived. Heat shock can induce apoptosis (41–43°C) or necrosis (>43°C) depending on the temperature that is applied (110). The immunogenicity of heat shock treated cancer cells largely resides within their ability to produce HSPs, such as HSP60, HSP70, and HSP90 (17, 32) (Table 1). These HSPs can function as chaperones for tumor antigens, facilitating their cross-presentation (17). Moreover, after recognition by their receptors (CD91, TLR2/4), these HSPs can instigate the attraction of neutrophils and monocytes and the activation of NK cells and DCs (111). These events are crucial for the initiation of tumor-specific immune responses. Independent of the induction of HSPs, heat shock treatment has also been shown to upregulate the transcription of specific tumor-associated antigens (34).

Co-incubation of heat-stressed apoptotic cancer cells with immature DCs resulted in the upregulation of DC maturation markers (CD40, CD80, and CD86) and higher IL-12 levels (32) (Table 1). Interestingly, splenocytes from mice immunized with heat-stressed apoptotic cancer cells got polarized toward a Th1 cytokine profile. Furthermore, DCs loaded with heat shock stressed melanoma cells can efficiently cross-prime tumor-antigen-specific CTLs both *in vitro* and *in vivo* (34). Of note, direct comparison of heat shock treated tumor lysate with freeze–thaw tumor lysate in a DC vaccine setup demonstrated a stronger tumor regression in favor of heat shock lysate in a mouse model for pancreatic cancer (Table 2). Again, this was associated with a stronger priming of tumor-specific CTL responses (17).

Dendritic cells loaded with heat shocked cancer cells have already been successfully applied in clinical practice in high-grade glioma patients (Table 2). Jie et al. recently published an open labeled non-randomized clinical trial in which 12 newly diagnosed glioblastoma patients received conventional therapy and 13 patients received additional DC vaccines loaded with heat shock treated autologous glioblastoma cells (67). The vaccinated patients had a significantly improved overall survival and progression-free survival. Interestingly, the proportions of peripheral CD4^+^ T cells, CD8^+^ T cells, and NK cells were significantly higher after DC vaccination in comparison to the control group. Moreover, increased levels of IFN-γ, IL-2, and IL-12 were measured in the sera of DC vaccinated patients.

**Figure 2:**
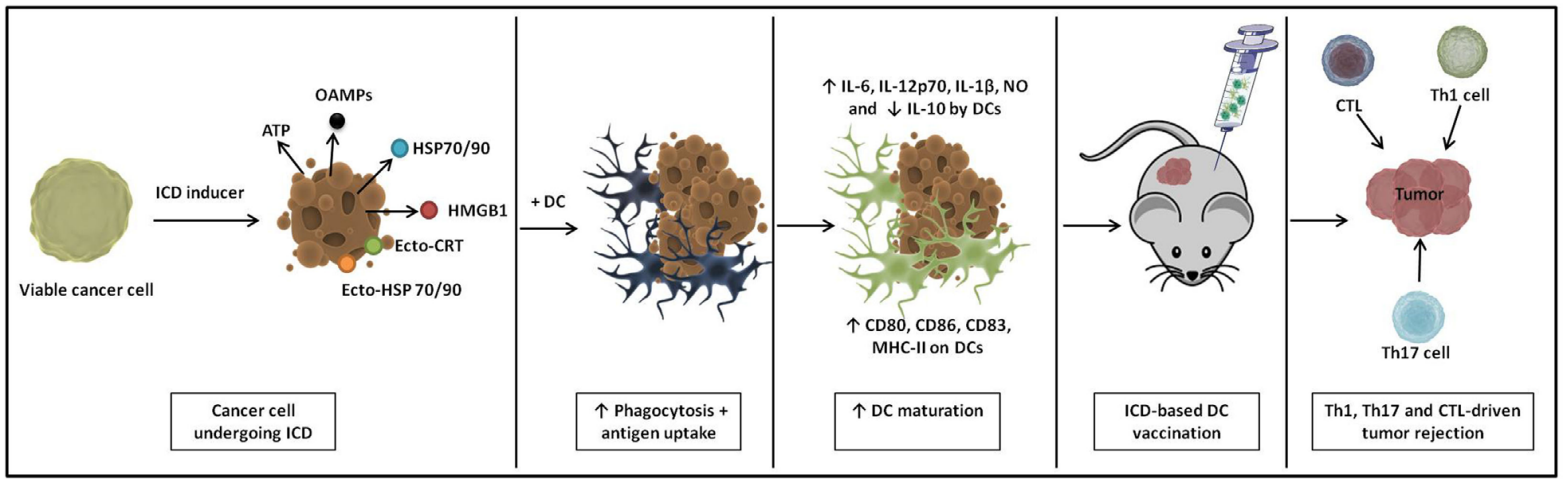
**A schematic representation of immunogenic cell death (ICD)-based DC vaccines**. ICD causes cancer cells to emit a spatiotemporally defined pattern of danger signals. Upon loading of these ICD-undergoing cancer cells onto DCs, they induce extensive phagocytosis and antigen uptake. Loaded DCs show enhanced phenotypic and functional maturation and immunization with these ICD-based DC vaccines instigates Th1-, Th17-, and cytotoxic T lymphocyte (CTL)-driven antitumor immunity *in vivo*.

All together, these data suggest that an immunogenic treatment of cancer cells can positively impact the potency of DCs interacting with them (Figure 1). In light of this finding, the relatively new concept of ICD of cancer cells can be considered a promising strategy for loading DC-based anticancer vaccines, potentially giving rise to a next generation of potent Th1-driving DC vaccines (111, 112) (Figure 2).

### Inducers of Immunogenic Cell Death

Immunogenic cell death is a cell death regimen that is associated with the spatiotemporally defined emission of immunogenic DAMPs that can trigger the immune system (20, 21, 113). ICD has been found to depend on the concomitant induction of reactive oxygen species (ROS) and activation of endoplasmatic reticulum (ER) stress (111). Besides the three DAMPs that are most crucial for ICD (ecto-CRT, ATP, and HMGB1), other DAMPs such as surface-exposed or released HSPs (notably HSP70 and HSP90) have also been shown to contribute to the immunogenic capacity of ICD inducers (20, 21). The binding of these DAMPs to their respective immune receptors (CD91 for HSPs/CRT, P2RX7/P2RY2 for ATP, and TLR2/4 for HMGB1/HSP70) leads to the recruitment and/or activation of innate immune cells and facilitates the uptake of tumor antigens by antigen-presenting cells and their cross-presentation to T cells eventually leading to IL-1β-, IL-17-, and IFN-γ-dependent tumor eradiation (22). This *in vivo* tumor rejecting capacity induced by dying cancer cells in the absence of any adjuvant, is considered as a prerequisite for an agent to be termed an ICD inducer. Recently, a classification system for ICD inducers was proposed based on whether an ICD inducer triggers apoptotic cell death as a consequence of direct action at the ER (Type II ICD inducer), or whether it initiates both ER stress-dependent danger signaling and apoptosis through divergent mechanisms (Type I ICD inducer) (111).

Although the list of ICD inducers is constantly growing (113), only few of these immunogenic modalities have been tested in order to generate an immunogenic tumor cell cargo to load DC vaccines (Tables 1 and 2). Figure 2 schematically represents the preparation of ICD-based DC vaccines and their putative modes of action.

#### Radiotherapy

Ionizing X-ray or γ-ray irradiation exerts its anticancer effect predominantly via its capacity to induce DNA double-strand breaks leading to intrinsic cancer cell apoptosis (114). The idea that radiotherapy could also impact the immune system was derived from the observation that radiotherapy could induce T-cell-mediated delay of tumor growth in a non-irradiated lesion (115). This abscopal (ab-scopus, away from the target) effect of radiotherapy was later explained by the ICD-inducing capacity (116). Together with anthracyclines, γ-irradiation was one of the first treatment modalities identified to induce ICD. Although this type I ICD inducer is known to induce ROS, its ER stress-inducing capability remains largely unexplored (111). The DAMPs that are induced following radiotherapy treatment of cancer cells include the exposure of ecto-CRT (23, 24, 35) and ecto-HSP70 (36), and the release of HMGB1 (33, 35) (Table 1). Irradiated B16 melanoma cells have been shown to be efficiently phagocytosed by DCs and to induce phenotypic DC maturation (15, 37). In addition, human DCs pulsed with irradiated tumor cells could efficiently stimulate antigen-specific CTL responses (40) (Table 1). Furthermore, mice immunized with DCs loaded with irradiated cancer cells could efficiently suppress tumor growth following inoculation with live syngeneic tumor cells in multiple preclinical cancer models (15, 40). In this setting, splenocytes from vaccinated animals could efficiently prime CD4^+^ and CD8^+^ T cells and exerted antigen-specific cytolytic activity (15) (Table 2).

Dendritic cell vaccines exposed to irradiated cancer cells have also been successfully implemented in clinical practice in melanoma and HGG patients (8, 69, 70) (Table 2). Cho et al. have shown that the implementation of DC vaccines loaded with irradiated autologous tumor cells in the conventional treatment regimen of newly diagnosed glioblastoma patients could significantly prolong the median overall survival (by more than 15 months) as compared to a control group receiving solely conventional treatment (69). Interestingly, the group of Di Nicola reported that vaccination with DCs loaded with dying autologous tumor cells after exposure to a cell death protocol consisting of heat shock, γ-ray, and UV ray could elicit clinical responses in 6 out of 18 relapsed B-cell lymphoma patients (117). Later, they showed the impaired ability of the neoplastic cells used to vaccinate non-responders to undergo ICD upon exposure to the cell death protocol (57). Importantly, they revealed a positive association between the extent of CRT and HSP90 surface expression in the DC antigenic cargo and the clinical and immunological responses achieved (57).

#### Shikonin

The phytochemical shikonin, a major component of Chinese herbal medicine, is known to inhibit proteasome activity. It serves multiple biological roles and can be applied as an antibacterial, antiviral, anti-inflammatory, and anticancer treatment. The latter application has been shown to yield responsiveness in late-stage lung cancer patients (118). Apoptotic cell death elicited by this type I ICD inducer can be inhibited by anti-oxidants, suggesting a role of shikonin-induced ROS (119, 120). The link between shikonin treatment and ER stress is not evidenced yet. The ICD that is induced in shikonin-treated cancer cells is characterized by the early induction of HSP70, HSP90, GRP78, and HMGB1 (41) (Table 1). Importantly, shikonin treatment could significantly improve the survival of mice bearing P388 leukemia and this antitumor effect of shikonin was less pronounced in immunodeficient mice (120). Moreover, the tumor lysate from shikonin-treated B16 cells could enhance phenotypic and functional DC maturation and differentiation of Th1 and Th17 cells, two important features of ICD-associated antitumor immunity (41) (Table 1). Additionally, curative vaccination of B16 melanoma-inoculated mice with shikonin-lysate-loaded DCs could delay tumor growth (41). This was associated with increased cytolytic activity of splenocytes on target tumor cells (Table 2). Although shikonin is administered to breast cancer patients for observational application (NCT01287468), clinical experience evaluating shikonin-lysate-loaded DC vaccines is unfortunately still lacking (Table 2).

#### High-Hydrostatic Pressure

High-hydrostatic pressure (HHP) is an established method to sterilize pharmaceuticals, human transplants, and food. HHP between 100 and 250 megapascal (MPa) has been shown to induce apoptosis of murine and human (cancer) cells (121–123). While DNA damage does not seem to be induced by HHP <1000 MPa, HHP can inhibit enzymatic functions and the synthesis of cellular proteins (122). Increased ROS production was detected in HHP-treated cancer cell lines and ER stress was evidenced by the rapid phosphorylation of eIF2α (42).

The anticancer activity of HHP was already demonstrated more than four decades ago in bladder cancer patients (124). Later, preclinical experiments demonstrated *in vivo* immunogenicity of HHP-treated cancer cells in the B16 melanoma model and the 3LL-D122 lung metastasis model (125, 126). Subsequently, it was shown that HHP-treated mammalian cancer cell lines undergoing apoptosis can release HSP70 and HMGB1, while retaining their immunogenicity *in vivo* (127). Very recently, Fucikova and colleagues have shown the ability of HHP to induce prototypical ICD in human prostate and ovarian cancer cell lines and in acute leukemia cells (42). HHP treatment induced the rapid expression of ecto-HSP70, ecto-HSP90, and ecto-CRT and the release of HMGB1 and ATP (Table 1). Interestingly, HHP-treated cancer cells were rapidly phagocytosed by DCs and induced the upregulation of CD83, CD86, and HLA-DR, and the release of pro-inflammatory cytokines (Table 1). This led to the stimulation of high numbers of tumor-specific T cells without inducing Tregs. Hence, all ICD-associated molecular criteria are fulfilled for HHP. This group is currently testing the *in vivo* immunogenicity of HHP killed tumor cells in prophylactic and curative murine vaccination settings (Table 2). Moreover, they have initiated multiple clinical trials to evaluate the potential of DC vaccines loaded with HHP-treated cancer cells in ovarian and prostate cancer patients (71).

#### Oncolytic Viruses

Oncolytic viruses are self-replicating, tumor selective virus strains that can directly lyse tumor cells. Over the past few years, a new oncolytic paradigm has risen; entailing that, rather than utilizing oncolytic viruses solely for direct tumor eradication, the cell death they induce should be accompanied by the elicitation of antitumor immune responses to maximize their therapeutic efficacy (128). One way in which these oncolytic viruses can fulfill this oncolytic paradigm is by inducing ICD (128).

Thus far, three oncolytic virus strains can meet the molecular requirements of ICD; coxsackievirus B3 (CVB3), oncolytic adenovirus and Newcastle disease virus (NDV) (Table 1) (113). Infection of tumor cells with these viruses causes the production of viral envelop proteins that induce ER stress by overloading the ER. Hence, all three virus strains can be considered type II ICD inducers (113). While CVB3 and oncolytic adenoviruses induce the surface expression of CRT, followed by the release of ATP and the passive release of HMGB1 in apoptotic tumor cells (in non-small cell lung carcinoma and adenocarcinoma cells, respectively) (43, 44), NDV induces necroptosis accompanied by the surface exposure of ATP and the post-necroptotic release of HMGB1 in GL261 glioma cells, with no contribution of ATP (Table 1) (45). In addition, NDV-infected GL261 cells upregulated the expression of the PMEL17 tumor antigen (45).

Intratumoral administration of CVB3 in nude mice resulted in the marked infiltration of NK cells, macrophages, granulocytes, and mature DCs into the tumor tissue (Table 1) (44). Tumor-infiltrating DCs expressed significantly higher levels of costimulatory molecules CD80 and CD86, as well as the lymph node homing receptor CCR7 (44). CD40-ligand encoding oncolytic adenoviruses have also been shown to facilitate the recruitment of DCs to the tumor tissue, this way entailing efficient Th1 and CD8^+^ CTL responses (Table 1) (43). Measles virus is another oncolytic virus that requires further investigation. Although extensive analysis of *in vitro* ICD determinants is lacking for this virus (only the release of HMGB1 has been documented), DCs exposed *in vitro* to measles-virus treated melanoma cells showed increased CD80 and CD86 expression levels (Table 1) (46). This resulted in the efficient priming of melanoma-specific cell killing by IFN-γ producing CD8^+^ T cells. Moreover, in terms of priming these melanoma-specific CTL responses, measles virus-infected melanoma cells constituted more effective tumor lysates (also termed oncolysates) for loading of DCs than uninfected melanoma cell lysates (46). The DC stimulatory capacity of NDV-derived oncolysates has already been demonstrated more than a decade ago by Schirrmacher et al. (47). DCs derived from breast cancer patients pulsed with NDV-oncolysates showed increased expression of costimulatory molecules in comparison to DCs loaded with tumor lysate from non-infected breast carcinoma cells (Table 1) (47). In addition, NDV-oncolysate-loaded DCs were more effective in stimulating bone-marrow-derived reactive memory T cells *in vitro* (47).

Oncolytic viruses hold great potential for application in ICD-based DC vaccines given their potential to elicit several ICD-related DAMPs. Furthermore, these viruses might directly affect DC maturation and activation through interaction with pathogen recognition receptors on the tumor cells. This way, biological oncolysates may render the use of an artificial maturation cocktail otiose. Unfortunately, there are no preclinical *in vivo* data available yet to evince the efficacy of DC vaccines loaded with immunogenic oncolysates (Table 2). Nevertheless, several studies have documented the beneficial effect of intratumoral application of oncolytic viruses in combination with tumor-directed systemic DC vaccinations (72, 73). Very recently, Schirrmacher et al. disclosed a case report of a breast cancer patient with liver metastasis that was treated with local hyperthermia, intravenously administered NDV, and subcutaneous vaccination with DCs loaded with NDV-infected breast cancer cells (oncolysate) (74). This combination therapy led to long-lasting tumor-specific memory T cell responses and stable disease for more than 66 months in this particular patient. The use of autologous DCs loaded with NDV-mediated oncolysate is licensed by the Paul Ehrlich Institute to the Immunologic-Oncologic Centre Cologne (IOZK) since May 2015.

Of note, in October 2015, the FDA approved the first oncolytic virus, Imlygic (a genetically modified live oncolytic herpes virus) for the treatment of melanoma lesions in the skin and lymph nodes. This FDA approval should facilitate the approval of other oncolytic viruses as well as the application of oncolysates in DC vaccine settings.

#### Photodynamic Therapy

Photodynamic therapy (PDT) is an established, minimally invasive anticancer treatment modality. It has a two-step mode of action involving the selective uptake of a photosensitizer by the tumor tissue, followed by its activation by light of a specific wavelength. This activation results in the photochemical production of ROS in the presence of oxygen (129–131). One attractive feature of PDT is that the ROS-based oxidative stress originates in the particular subcellular location where the photosensitizer tends to accumulate, ultimately leading to the destruction of the tumor cell (132). PDT-based antitumor effects are multifactorial and depend on its abilities to damage the tumor vasculature, directly kill tumor cells, exert cytotoxic effects toward tumor-infiltrating immune cells, and recruit and activate immune cells that can initiate adaptive antitumor immune responses (131).

Increasing preclinical information is available regarding the impact of PDT on the immune system. Recent studies have demonstrated that PDT can effectively generate several DAMPs. HSP70, the best studied DAMP associated with PDT, is exposed on the surface of cancer cells treated with photofrin-PDT, 5-Aminolevulinic acid (5-ALA)-PDT, and Foscan-PDT (51, 133, 134). Of note, the uptake of tumor antigens and DC maturation induced by 5-ALA-PDT treated GBM spheroids were inhibited when HSP70 was blocked (133). Later, it was reported that photofrin-PDT also promotes the early/mid-apoptotic surface expression of CRT and the post-apoptotic release of HMGB1 (52) (Table 1). Very recently, the DAMPs profile induced by Rose Bengal Acetate (RBAc)-based PDT was unraveled. RBAc-photosensitized apoptotic/autophagic Hela cells were found to expose and/or release ATP, HSP70/90, HMGB1, and CRT (135). In terms of its immunogenicity, hypericin can be considered the best studied photosensitizer. Recently, hypericin-PDT became the first PDT modality capable of inducing prototypical ICD in cancer cells (20, 48, 49, 111). Hypericin localizes predominantly in the ER and upon irradiation it causes photo-oxidative ER stress, making hypericin-PDT the only known modality able to induce ICD through focused ROS-based ER stress (Type II ICD inducer), eventually culminating in mitochondrial apoptosis (49, 136). In the pre-apoptotic stage, it induces the active emission of three crucial ICD-associated DAMPs, i.e., ecto-CRT, ecto-HSP, and secreted ATP (at a faster rate than what was previously published for these DAMPs), followed by the passive release of HSP70 and HMGB1 (48, 49) (Table 1). Interestingly, this ICD-subroutine was more effective in comparison to chemotherapy- or radiotherapy-induced ICD (48, 49).

The immunogenic features of Hyp-PDT–treated cancer cells have also been confirmed by *ex vivo* and *in vivo* experiments (Tables 1 and 2). Hyp-PDT-treated cancer cells form a productive interface with DCs in terms of phagocytosis (CRT-dependent) and maturation (49) (Table 1). More specifically, the interacting DCs exhibit functional stimulation (NO^high^, IL-10^absent^, IL-6^high^, IL-1β^high^, and IL-12p70^median^) and phenotypic maturation (CD80^high^, CD83^high^, CD86^high^, and MHC-II^high^) (49, 53). Moreover, these immunogenic and fully mature DCs induce the clonal expansion of human IFN-γ producing CD4^+^ and CD8^+^ T cells (53, 54). Consequently, this *in vitro* antitumor immunity induced by Hyp-PDT-induced ICD led to the efficient rejection of murine tumors *in vivo* in the absence of any adjuvants (both in prophylactic and curative vaccination models) (49, 137). Besides hypericin-based PDT, photofrin-based PDT is to date the only PDT modality that is capable to fulfill this critical *in vivo* requirement for ICD characterization. Here, curative immunization with benzoporphyrin-based PDT-treated squamous cell carcinoma cells constituted a potent anticancer vaccine in this poorly immunogenic model (56).

Importantly, inoculation of mature DCs in PDT-treated tumors resulted in the cytolytic activation of T cells and NK cells, leading to effective tumor eradication (75). Moreover, DC vaccines loaded with PDT-induced tumor lysates have been shown to cure fully established solid non-orthotopic tumors. This was associated with enhanced CTL responses and Th1 immunity (138) (Table 2). These data already suggest the clinical potential of PDT-based DC vaccines. In this regard, Hyp-PDT-induced ICD-based DC vaccines are currently being tested in a preclinical model for ovarian cancer by Baert et al. (personal communication). Unfortunately, there are no clinical data available yet reporting the use of PDT-based DC vaccines.

### Combinatorial Regimens

In DC vaccine settings, cancer cells are often not killed by a single treatment strategy but rather by a combination of treatments. In some cases, the underlying rationale lies within the additive or even synergistic value of combining several moderately immunogenic modalities. The combination of radiotherapy and heat shock has, for instance, been shown to induce higher levels of HSP70 in B16 melanoma cells than either therapy alone (16). In addition, a combination therapy consisting of heat shock, γ-irradiation, and UV irradiation has been shown to induce higher levels of ecto-CRT, ecto-HSP90, HMGB1, and ATP in comparison to either therapy alone or doxorubicin, a well-recognized inducer of ICD (57). Besides, the sequence of the applied methodologies seems to matter. The application of radiotherapy prior to freeze–thaw cycles was recently shown to negatively impacted the survival of high-grade glioma-bearing mice (in comparison to freeze–thaw cycles followed by X-ray irradiation) in the context of DC-based immunotherapy (18). A second rationale for combining several cell killing methodologies is to meet the clinical requirement of reaching 100% cancer cell death (14). Subcutaneous injection of irradiated tumor cells has, for instance, induced subcutaneous tumor growth in one glioblastoma patient (139). In general, most single treatment modalities discussed in this review cannot meet this requirement, postulating their combination with other (potentially less immunogenic) cell death modalities. In view of this, preclinical testing should always consider the most clinically relevant version of the vaccine.

## Concluding Remarks

Triggering antitumor immune responses is an absolute requirement to tackle metastatic and diffusely infiltrating cancer cells that are resistant to standard-of-care therapeutic regimens. ICD-inducing modalities, such as PDT and radiotherapy, have been shown to be able to act as *in situ* vaccines capable of inducing immune responses that caused regression of distal untreated tumors. Exploiting these ICD inducers and other immunogenic modalities to obtain a highly immunogenic antigenic tumor cell cargo for loading DC vaccines is a highly promising application. In case of the two prominent ICD inducers, Hyp-PDT and HHP, preclinical studies evaluating this relatively new approach are underway and HHP-based DC vaccines are already undergoing clinical testing. In the preclinical testing phase, more attention should be paid to some clinically driven considerations. First, one should consider the requirement of 100% mortality of the tumor cells before *in vivo* application. A second consideration from clinical practice (especially in multi-center clinical trials) is the fact that most tumor specimens arrive in the lab in a frozen state. This implies that a significant number of cells have already undergone non-immunogenic necrosis before the experimental cell killing strategies are applied. In case of ICD inducers, this could potentially hamper the immunogenicity of the tumor cells as these modalities mainly rely on active danger signaling pathways. Finally, for a more clinically relevant evaluation of the effect of immunogenic DC vaccines on tumor cell stromal interactions, orthotopic tumor inoculation should be applied. As tumor cells are implanted in the anatomically appropriate location, orthotopic tumors reflect the clinical situation (e.g., the tumor microenvironment) much better than conventional subcutaneous non-orthotopic models.

Even the most potent active immunotherapeutic strategies such as (ICD-based) DC vaccines will, however, be hampered by the presence of immunomodulatory immune checkpoint molecules (such as PD-1 and CTLA-4) that inhibit cytotoxic immune responses or even induce immune tolerance. The development of drugs that can unleash these inhibitory molecules has become one of the most active areas in oncology. This creates the opportunity to combine checkpoint inhibitors with DC-based immunotherapy. The synergistic action of a CTLA-4 blocking Ab (tremelimumab) in combination with DC therapy has already been demonstrated in advanced melanoma patients and several other trials evaluating this approach are on the horizon (6, 140, 141).

We believe that the specialty of DC-based immunotherapy is considerably moving forward by focusing on developing more immunogenic Th1-driving vaccines, such as ICD-based DC vaccines. Moreover, the combination of ICD-based DC vaccines with checkpoint inhibitors or other drugs that can inhibit the severe tumor-induced immune suppression might be able to reveal the full efficacy of DC-based immunotherapy for cancer.

## Author Contributions

LV did the literature study, data collection, and wrote the manuscript. SV provided senior supervision, helped in writing, and critically revised the manuscript. All the other co-authors have substantially contributed to the design of the work. All co-authors critically revised the manuscript and approved the final manuscript. All co-authors can be considered accountable for all aspects of the work.

## Conflict of Interest Statement

The authors declare that the research was conducted in the absence of any commercial or financial relationships that could be construed as a potential conflict of interest.

## Abbreviations

CD, cluster of differentiation; CRT, calreticulin; CVB3, coxsacievirus B3; DAMP, damage-associated molecular pattern; DC, dendritic cell; ER, endoplasmic reticulum; HHP, high-hydrostatic pressure; HMGB1, high-mobility group box 1; HSP, heat shock protein; Hyp, hypericin; ICD, immunogenic cell death; NDV, Newcastle disease virus; OAMP, oxidation-associated molecular pattern; PDT, photodynamic therapy; ROS, reactive oxygen species; TLR, Toll-like receptor; UV, ultraviolet.
